# Linear relationships between aboveground biomass and plant species diversity during the initial stage of degraded grassland restoration projects

**DOI:** 10.1002/ece3.70128

**Published:** 2024-08-12

**Authors:** Chun‐Jing Wang, Shan‐Feng Huang, Chu‐Ping Wu, Gai‐Ni Wang, Lei Wang, Yong‐Kun Zhang, Ji‐Zhong Wan

**Affiliations:** ^1^ Sichuan Academy of Forestry Chengdu China; ^2^ State Key Laboratory of Plateau Ecology and Agriculture Qinghai University Xining China; ^3^ Climate Bridge Ltd (Shanghai) Shanghai China; ^4^ Zhejiang Academy of Forestry Hangzhou China; ^5^ College of Wildlife and Protected Area Northeast Forestry University Harbin China; ^6^ Key Laboratory of Mountain Surface Processes and Ecological Regulation, Institute of Mountain Hazards and Environment Chinese Academy of Sciences Chengdu China

**Keywords:** biomass, carbon sequestration project, ecological restoration, land degradation, plant diversity, weed invasion

## Abstract

The relationship between aboveground biomass and plant diversity has been extensively examined to understand the role of biodiversity in ecosystem functions and services. Degraded grassland restoration projects can enhance carbon sequestration. However, the relationship between biomass and diversity remains one of the most actively debated topics regarding grassland ecosystems in degraded grassland restoration projects. We speculated that establishing the linear relationships between aboveground biomass and plant species diversity could contribute to enhancing the efficacy of degraded grassland restoration projects. This study sought to determine whether these relationships were linear during the initial stages of the restoration projects of degraded grasslands in Xing'an League, China. The investigations were based on an examination of seventy‐six 1 × 1 m^2^ plots distributed among 15 areas in which the degraded grassland was at the initial stages of restoration. To quantify the species diversity of the degraded grassland communities, we used the species richness, Shannon–Wiener, inverse Simpson's reciprocal, and Pielou's evenness indices. Our analyses revealed that aboveground biomass had clear positive linear relationships with species richness during the initial stages of degraded grassland restoration. However, there were less pronounced associations with species diversity as assessed using the Shannon and inverse Simpson indices, based on regression models. Furthermore, weed biomass was found to have significant negative effects on species richness and Pielou's evenness. The weak linear relationship between aboveground biomass and species richness could be ascribed to an increase in weed biomass. We concluded that aboveground biomass and plant species diversity could be enhanced during the initial stages of degraded grassland restoration projects and suggest that the extent of weed biomass could serve as a key indicator of the efficacy of restoration from the perspective of plant species diversity and aboveground biomass in carbon sequestration projects.

## Graphical abstract

We assessed linear relationships between aboveground biomass and plant species diversity. Our findings indicated that both aboveground biomass and plant species diversity could be effectively restored during the initial stages of degraded grassland restoration projects. We believe that our study will contribute to enhancing the successional development of once degraded grassland landscapes.

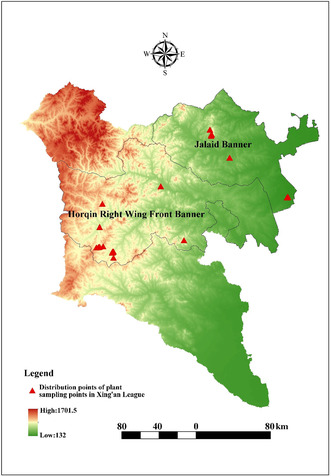

## 1 INTRODUCTION

The relationship between aboveground biomass and plant diversity has been the focus of extensive ecological research, the findings of which have yielded valuable insights into the role of biodiversity in ecosystem performance and have generated considerable debate (Brun et al., 2019; Grace et al., 2016; Waide et al., 1999). Although canonical bivariate biomass–diversity relationships have been established to occur widely in ecological systems, the relationships between biomass and species diversity are among the most debated subjects in the history of plant ecology (Adler et al., 2011; Guo, 2007; Oba et al., 2001). Numerous studies (e.g., Brun et al., 2019; Guo, 2007; Oba et al., 2001; Waide et al., 1999) have shown that biomass–diversity relationships may be positive or bivariate depending on habitat type, and Grace et al. (2016) proposed that developing local diversity is influenced by a combination of habitat heterogeneity and disturbance. Hence, there is a strong likelihood that the degradation of areas and habitats will lead to changes in biomass–diversity relationships.

Recently, with a view toward carbon sequestration, there has been a considerable worldwide growth in the number of projects designed to restore degraded grasslands, which can make a substantial contribution to carbon sequestration, enhancing environmental management and carbon market development (https://registry.verra.org/). Moreover, grassland restoration and maintenance, particularly during the initial stages of restoration projects, play a key role in addressing global issues such as the biodiversity conservation and adaptation to climate change (De Deyn et al., 2011; Lyons et al., 2023). During the initial stages of restoration projects, measures such as study site assessments identify specific site characteristics and define the needs and goals for restoration, determining the likelihood of achieving grassland restoration objectives (Ehrenfeld, 2000; Gann et al., 2019; Palik et al., 2000). In this regard, diversity and biomass are considered two key variables of grassland ecosystem functioning and services during the initial periods of grassland restoration projects that contribute to the efficacy of degraded grassland restoration (De Deyn et al., 2011; Guo, 2007; Pastorok et al., 1997). High levels of aboveground biomass and plant diversity, which have a reciprocal influence, should appear during the initial phase of restoration projects (Guo, 2007; Martin et al., 2005). It has been predicted that such increases in biomass and diversity would enable carbon sequestration projects to reach the requisite climate, community, and biodiversity (CCB) standards that aid local communities and biodiversity, delivering lower carbon offsets (Bastias et al., 2021). Hence, we believe that the linear relationship between the aboveground biomass and plant diversity contributes to the efficacy of degraded grassland restoration projects.

The challenge in degraded grassland restoration projects is to reduce the cover of invasive weed species while retaining existing native species and increasing their biomass and diversity (Funk et al., 2008; Guo et al., 2018). From the perspectives of aboveground biomass and plant diversity, there are considerable challenges regarding the efficacy of grassland restoration projects, such as weed invasion (Allen & Meyer, 2014; Humphries et al., 2021; Török et al., 2021). Highly competitive invasive weed species are often a major impediment to the successful establishment and/or subsequent survival of native plant species in degraded grasslands, and site‐specific management is essential to ensure the ongoing suppression of unwanted weed species (Humphries et al., 2021; Weidlich et al., 2020). Indeed, the outcome of restoration initiatives is often determined by the reestablishment of invasive weeds despite management efforts (Humphries et al., 2021; Török et al., 2021; Weidlich et al., 2020). Accordingly, measures should be taken to control or eliminate weeds and undesired vegetation that may outcompete the native plant species (Assis et al., 2021; Török et al., 2021; Weidlich et al., 2020). Hence, to enhance the efficacy of grassland restoration initiatives, it is necessary to understand the effects of invasive weeds on the relationship between aboveground biomass and plant diversity when assessing the recovery of these parameters during the initial stages of restoration.

We predicted that the initial phase of grassland restoration would be characterized by high levels of aboveground biomass and plant diversity and accordingly reasoned that determining the linear relationships between these two parameters would provide valuable insights into the processes underlying the initial stages of vegetation recovery (Brun et al., 2019; Gann et al., 2019; Grace et al., 2016; Waide et al., 1999). Thus, the aboveground biomass increases in response to increased plant diversity. We reasoned that our assessment of the efficacy of grassland restoration should consider the influence of invasive weeds on the reestablishment of native vegetation. In this study, we used different species diversity indices to assess the recovery of degraded grassland areas in the Xing'an League in the northeastern region of the Inner Mongolia Autonomous Region, China. With respect to the initial restoration of degraded grasslands, we proposed the following three hypotheses: (1) the relationship between aboveground biomass and plant species diversity would be linear, (2) there would be differences in aboveground biomass and plant species diversity between weed‐invaded and non‐invaded plots, and (3) there would be significant relationships between weed biomass, aboveground biomass, and plant community diversity in weed‐invaded areas.

## 2 MATERIALS AND METHODS

### 2.1 Study region

Xing'an League is located at the southern foot of the Greater Xing'an Mountains within the Xinhua Xia tectonic mountain range in the northeast of the Inner Mongolia Autonomous Region, China. Xing'an borders Heilongjiang Province in the northeast, Jilin Province in the southeast, and Tongliao City, Xilingol League, and Hulunbuir City in the south, west, and north of Inner Mongolia, respectively (Jia et al., 2018). In the northwest, it shares a 126‐km border with Mongolia (Dong et al., 2018). From north to south and east to west, Xing'an League ranges over distances of 380 and 320 km, respectively, and spans corresponding latitudes and longitudes of 44°15′ N to 47°39′ N and 119°28′ E to 123°39′ E (Dong et al., 2018; Jia et al., 2018).

### 2.2 Fieldwork

Our fieldwork was undertaken over a 5‐year period, spanning the initial stages of restoration projects of degraded grasslands in the Xing'an League and based on the standardized methodological protocol proposed by the Dark Diversity Network (DarkDivNet; Pärtel et al., 2019). Under the degraded grassland restoration project of the Xing'an League, the Horqin Right Front Banner and Jalaid Banner had confirmed development areas of 635,000 mu. This project was verified using the CCB standards.

We investigated 15 study areas (encompassed within a circle of 10 km radius) that were similar in terms of environmental conditions (e.g., soil and climate) and covered a range of altitudes typical of the Xing'an League. Based on typical natural or semi‐natural target habitat types (e.g., semi‐natural and artificial grasslands), we selected the abovementioned 15 study areas for fieldwork. Within each study area, we selected one to three typical natural or semi‐natural target habitat types that were relatively common (i.e., we avoided very rare and extreme habitat types). We classified the 15 study areas into seven groups based on the level of disturbance.

**FIGURE 1:**
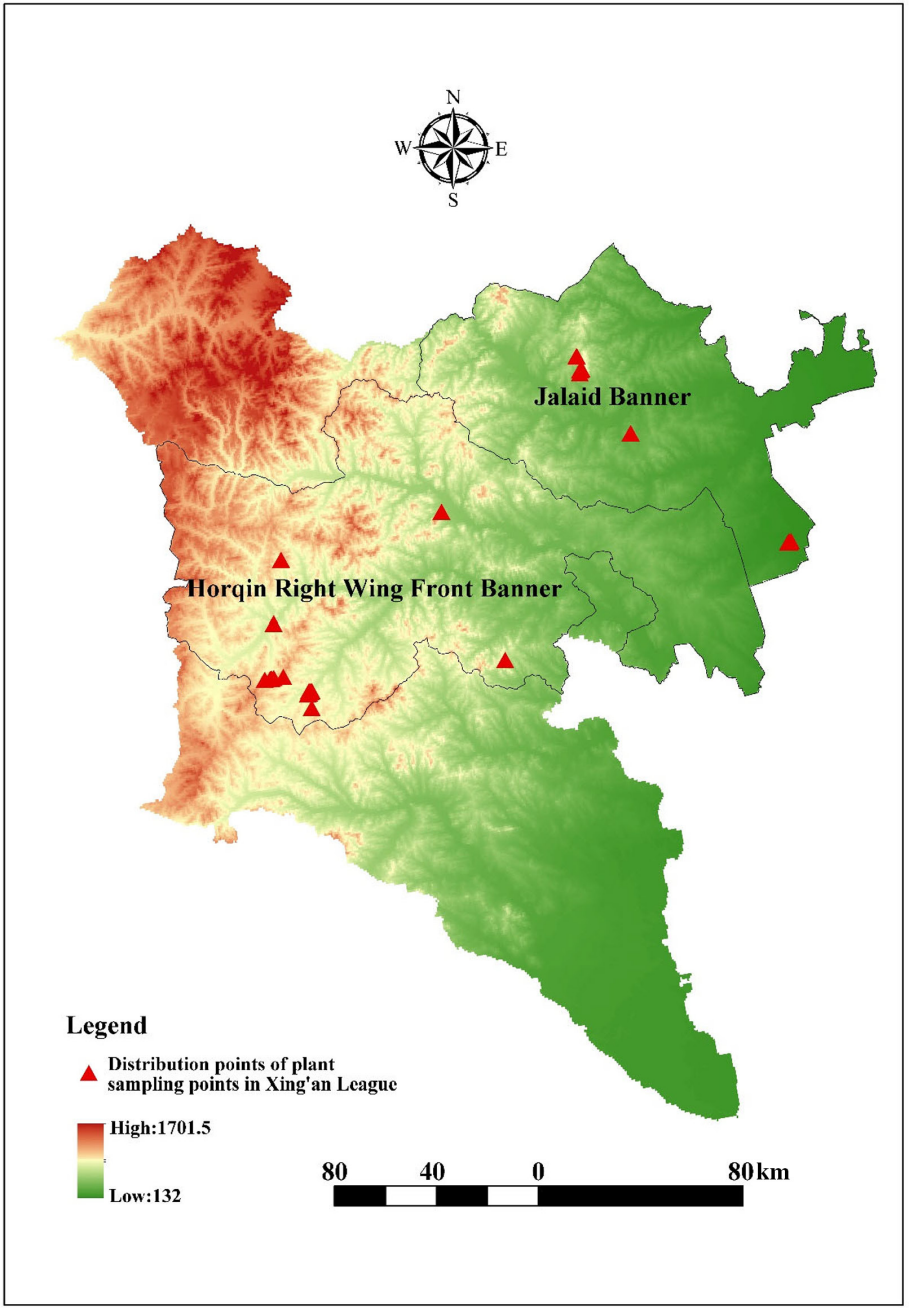
The distribution map of investigation plots for terrestrial plant communities in Xing'an League (blue line), China from Google Earth.

For each of these target habitats, we obtained samples from paired study sites defined by the level of anthropogenic disturbance (i.e., relatively natural vs. disturbed). We established five 1 × 1 m^2^ plots, including one core plot and four surrounding plots. The core plot was centered on the surrounding plots. The core plot should include species richness in each study area and a gradient of anthropogenic disturbances for vegetation in the surrounding plots. One or two of the four surrounding plots should include weed species if they occur in the study area within the target habitat types. In the Xing'an League, we established seventy‐six 1 × 1 m^2^ plots distributed in 15 study areas along an altitudinal gradient and conducted floristic surveys to determine plant composition during the initial‐stage grassland restoration from June to July 2023, as shown in Figure 1. For each of the surveyed sites, we recorded the following data: (1) geographical coordinates (latitude and longitude) at the center of the investigated sites, (2) altitude at the center of the sites, (3) vegetation type specification, (4) abundance of identified species, and (5) identity of vascular plant species following The Plant List (http://www.theplantlist.org; Pärtel et al., 2019).

### 2.3 Measurement of aboveground biomass

To determine the aboveground biomass, plants within each 1 × 1 m^2^ plot were cut at ground level, weighed immediately after cutting, and placed in labeled plastic bags. Biomass was determined for each plot on a dry matter basis by drying samples in a 60°C oven for 2 days until a constant weight had been obtained (Morais et al., 2021). Biomass data can be collected on an individual species basis as the total weight of the vegetation (Morais et al., 2021).

### 2.4 Measurements of plant species diversity

The abundance of each plant species in each plot was determined based on visual observations and the identification of cut specimens. To quantify the species diversity of the terrestrial plant community, we used the species richness (*S*), Shannon–Wiener (*H*), Simpson (*D*), and Pielou's evenness (*J*) indices derived as follows:

(1) S=n,

where *n* represents the total number of species observed in each investigated plot.

(2) $$H=-\sum_{i=1}^{s}p_{i}\;\ln\;p_{i},$$

(3) $$D=\sum_{i=1}^{s}p_{i}^{2},$$

(4) J=HlnS,

where *p_i_* represents the proportion of the total plant abundance of species *i* in each investigated site (Bandeira et al., 2013). Here, we used inverse *D* for further analysis.

### 2.5 Analyses

Prior to the analyses, the values obtained for biomass and all species diversity indices were log_10_ transformed. Initially, we used simple linear models in conjunction with the ordinary least squares (OLS) method to examine the relationships between aboveground biomass and the four assessed plant diversity indices (species richness, Shannon–Wiener, inverse Simpson, and Pielou's evenness). Thereafter, we performed analyses of variance to assess the differences in aboveground biomass and species richness, Shannon, Inverse Simpson, and Pielou's evenness index values recorded in paired weed‐invaded and non‐invaded plots. Finally, we used simple linear models to assess the relationships between weed biomass and aboveground biomass and species richness, Shannon, Inverse Simpson, Pielou's evenness index values, and the residuals of OLS based on plot data from the northeastern Inner Mongolia Autonomous Region. All statistical analyses were conducted using JMP 17.0 software (https://www.jmp.com/en_us/home.html) and “vegan” package in the R environment (https://www.r‐project.org/).

## 3 RESULTS

**FIGURE 2:**
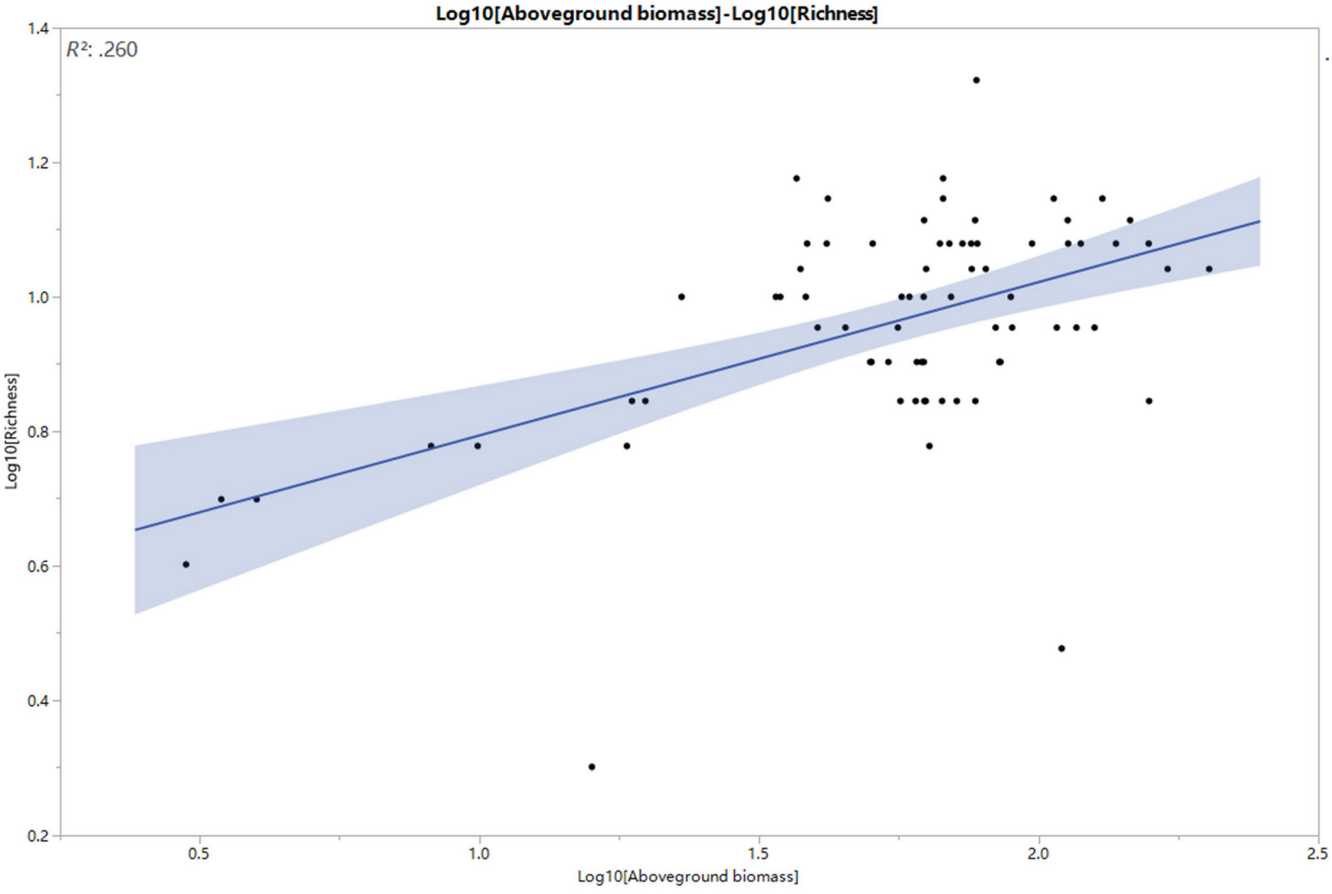
Regression lines for relationships of aboveground biomass with species richness for terrestrial plant communities in Xing'an League, China. The relationship was significant (*p* < .05). The shaded band is a pointwise 95% confidence interval on the fitted values (the line).

The ranges were 2–21 for species richness, 0.033–2.140 for Shannon–Wiener, 1.011–7.846 for the inverse Simpson, 0.047–0.937 for Pielou, and 2.988–202.137 g/m^2^ for aboveground biomass in plantation forests of carbon sequestration projects in Tianshui (Table 1). During the initial stages of degraded grassland restoration, the aboveground biomass had a clear positive linear relationship with species richness (Table 2; Figure 2). Comparatively, we detected less pronounced associations between biomass and the Shannon and inverse Simpson diversity indices, for which species diversity values were derived based on the regression models (Table 2).

**TABLE 1:** Summary of species diversity and biomass of terrestrial plant communities in Xing'an League, China.

	Mean	SD	Minimum	Maximum
Shannon	1.491	0.169	0.033	2.140
Richness	9.776	9.803	2.000	21.000
InverseSimpson	3.658	2.053	1.011	7.846
Pielou	0.666	0.025	0.047	0.937
Aboveground biomass (g/m^2^)	70.825	1637.742	2.988	202.137
Weed biomass (g/m^2^)	11.656	213.067	0.408	56.514

**TABLE 2:** Relationships of aboveground and weed biomass (g/m^2^) with species diversity based on the results of simple linear models.

	Aboveground biomass			Weed biomass		
	*R* ^2^	*p*‐value	Slope	*R* ^2^	*p*‐value	Slope
Shannon	.062	.031	0.154	.052	.362	0.044
Richness	.260	.000	0.228	.183	.077	−0.048
InverseSimpson	.037	.095	0.100	.039	.430	0.055
Pielou	.005	.544	0.032	.123	.153	0.065

**FIGURE 3:**
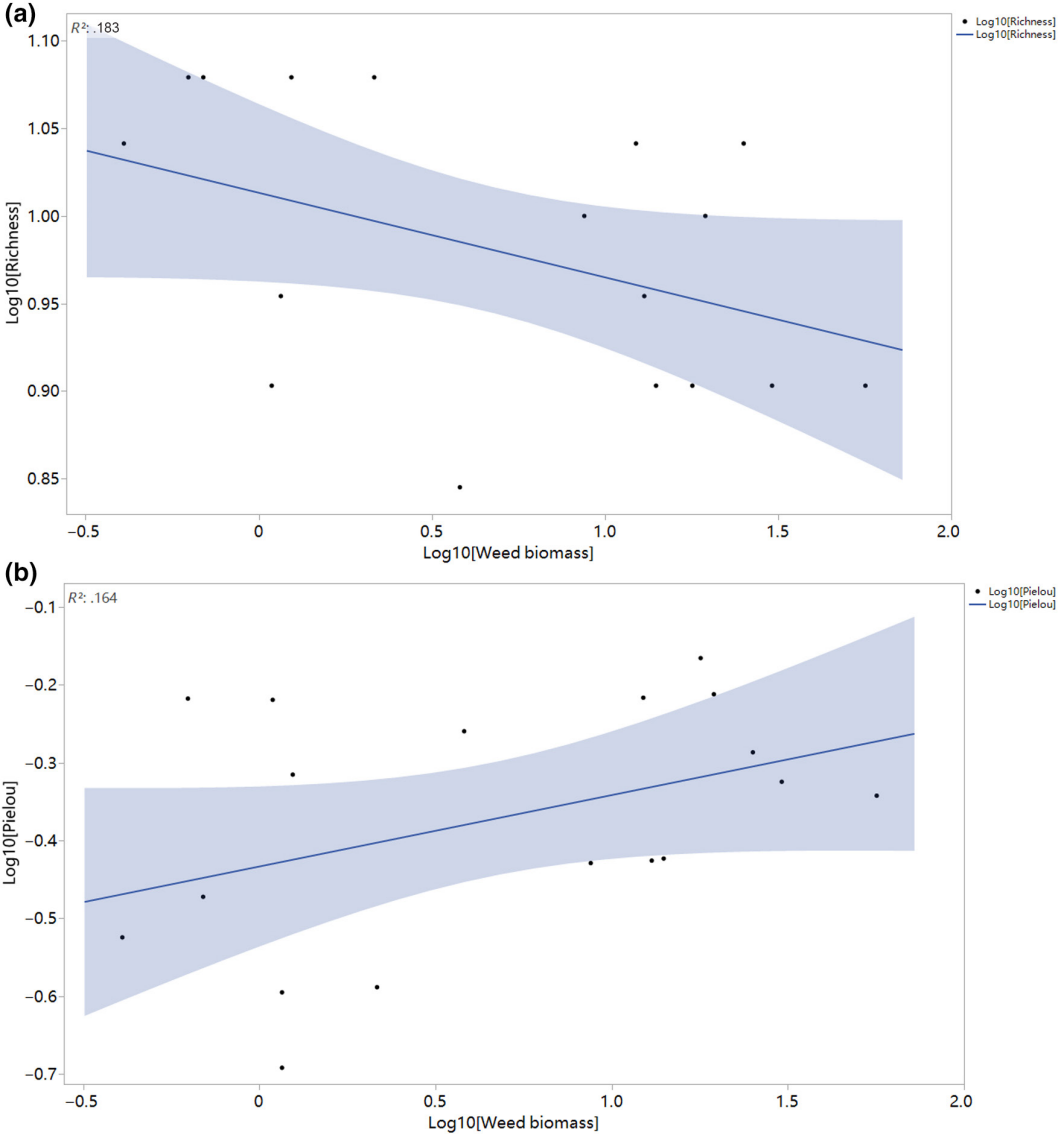
Effects of weed biomass on species richness for terrestrial plant communities in Xing'an League, China. The relationship was significant (*p* < .05). The shaded band is a pointwise 95% confidence interval on the fitted values (the line).

**FIGURE 4:**
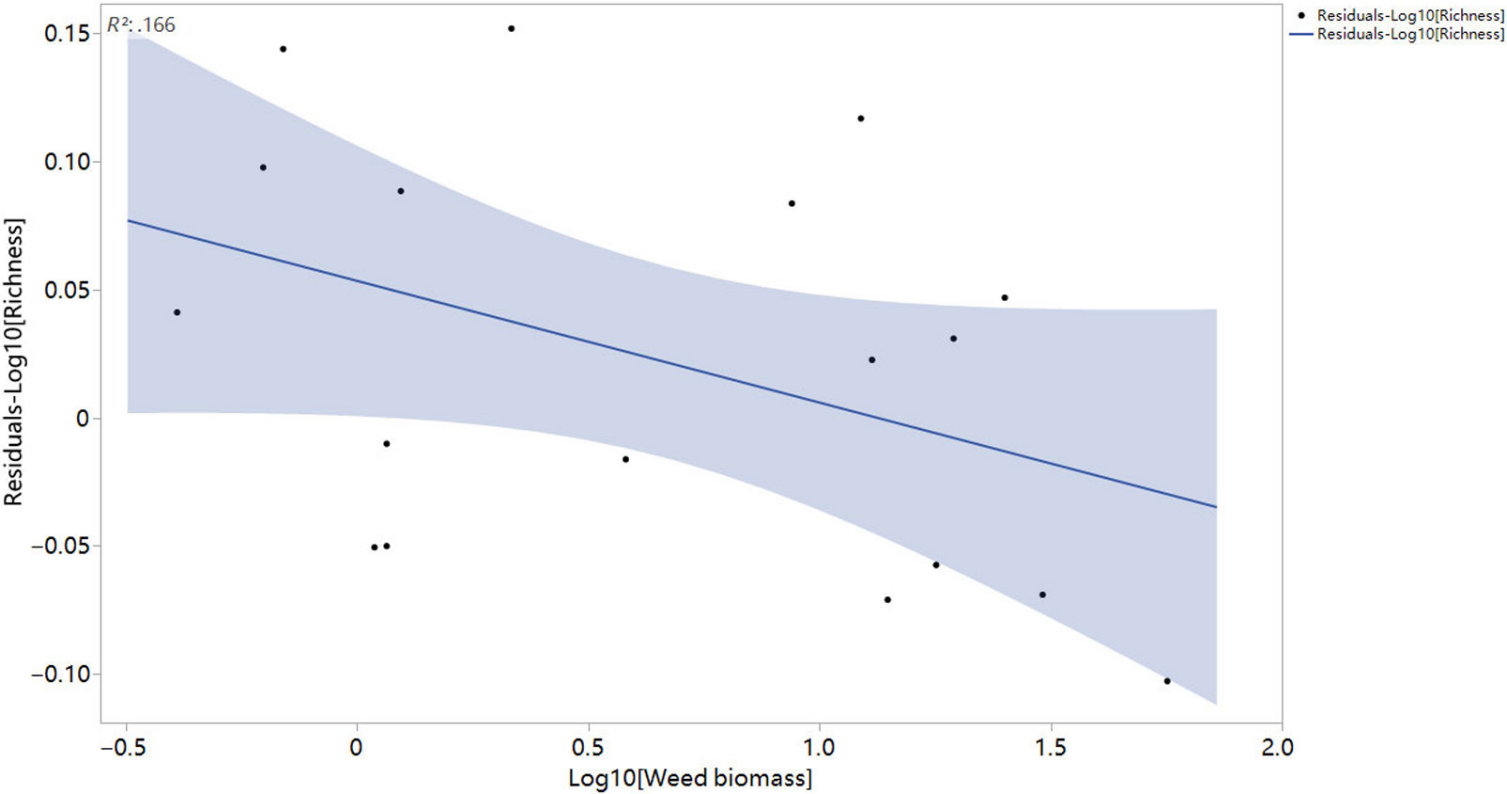
Effects of weed biomass on the residuals of the linear relationships between aboveground biomass and species richness for terrestrial plant communities in Xing'an League, China. The relationship was significant (*p* < .05). The shaded band is a pointwise 95% confidence interval on the fitted values (the line).

Weed invasions occurred in 18 plots in the study area. We recorded significantly lower Pielou evenness values in weed‐invaded and non‐invaded plots (Mean values: Non‐weed vs. Weed: −0.300 vs. −0.373; *p* < .05), and weed biomass was found to have significantly negative effects on species richness (*R* ^2^ = .183; *p* = .077) and Pielou's evenness (*R* ^2^ = .164; *p* = .096; Figure 3). Weed biomass was also shown to have a significant influence on the residuals of the linear relationships between aboveground biomass and species richness, with larger residuals recorded with increasing weed biomass (*R* ^2^ = .166; *p* = .093; Figure 4).

## 4 DISCUSSION

### 4.1 Linear relationships between aboveground biomass and plant species diversity

We found that during the initial stages of degraded grassland restoration, there was a positive linear relationship between aboveground biomass and species richness; however, the associations between biomass and Shannon, Inverse Simpson, and Pielou evenness indices were somewhat weaker. Based on their meta‐analysis, Adler et al. (2011) revealed the prevalence of hump‐shaped relationships between aboveground biomass and plant species richness, indicating that species richness is the highest at an intermediate level of aboveground biomass. The intermediate disturbance hypothesis, which predicts the highest diversity at intermediate disturbance levels, has been used to explain some of the more commonly observed patterns of species diversity (Gao & Carmel, 2020; Roxburgh et al., 2004). During the initial stages of the restoration of degraded grasslands, disturbance levels tend to be relatively high. Under these conditions, aboveground biomass may not reach its maximum potential, which may account for the relatively weak positive relationships with species diversity.

Restoration projects in Xing'an League include large areas of grazing land. Under high grazing pressure, species competition may be low for emerging species; however, in response to restoration, increasing levels of soil fertility would enhance the number of different plant species, which in turn would tend to increase the probability of encountering exceptionally productive species within herbaceous plant communities (Gao & Carmel, 2020; McIntyre & Lavorel, 2007; Osem et al., 2002; Ralphs, 2002). Under such conditions, species richness is directly associated with responses to abiotic (e.g., environmental filtering) and biotic (e.g., competitive exclusion) factors (Daleo et al., 2023; Myers & Harms, 2009; Wu et al., 2019). Ecological restoration creates conditions that facilitate the proliferation of grass plant communities, with early colonizers such as annual species potentially contributing to the enrichment of soil nutrients, whereas later‐occurring perennial species may provide cover and shade (Guo, 2007; Seabloom et al., 2015; Teague et al., 2011). Despite disturbance at the initial stage of restoration, with the continuation of the successional process, changes in resource availability may alter the balance in species composition and promote increases in species number; hence, our findings of a linear relationship between aboveground biomass and plant species richness during the initial phase of degraded grassland restoration.

### 4.2 Influence of invasive weeds

Our results revealed that Pielou's evenness was significantly lower in weed‐invaded and non‐invaded plots and that weed biomass had significant negative effects on species richness and Pielou's evenness. Weed invasion can reduce species richness and evenness in plant communities of degraded grasslands under restoration. As resident species disappear or become rare in degraded grasslands, new species enter ecosystems during the progression of restoration. In the grasslands of the Xing'an League, the plant communities that subsequently develop may differ from those that originally predominated prior to disturbance (Catford et al., 2012; Humphries et al., 2021; Renne & Tracy, 2013). The capacity of invasive weed species to establish and spread within grassland ecosystems is associated with their ability to competitively suppress resident species, which may only be successfully established under conditions where disturbances reduce competitive interference or priority effects in degraded grasslands (Buckley et al., 2007; Catford et al., 2012; Freitag et al., 2021). Hence, the reduction in species richness can be attributed to the combined effects of a reduction in resident species and an increase in weed species biomass.

Regarding our finding of a negative association between weed biomass and species evenness during the initial stages of degraded grassland restoration, it has been established that differences in habitat cover are important determinants of the influence of weed invasion on species evenness. In areas with low habitat cover, rare species are particularly susceptible to competition from invasive weeds, which increases their abundance of the remaining more common species (Bekker et al., 1997; Catford et al., 2012; Vila & Ibáñez, 2011). Given that in plant communities subjected to heavy weed invasion, the abundance of these invaders is generally higher than that of the most common native species, these communities are typically less diverse than those populating uninvaded habitats (Allen & Meyer, 2014; Humphries et al., 2021). During the initial stages of restoration, weed species can rapidly spread and establish habitats with sparse native vegetation coverage, facilitating the accumulation of large amounts of weed biomass (Freitag et al., 2021; Humphries et al., 2021).

Our residual results indicated the relatively weak relationship between the aboveground biomass and species richness could be attributed to weed biomass. In response to weed invasion, a reduction in the richness of rare and common species can contribute to changes in population growth (i.e., biomass) and size (i.e., abundance; Hejda et al., 2009; Catford et al., 2012). Increases in weed biomass can increase the probability of native plants becoming locally extinct due to deterministic and stochastic processes associated with lower local population sizes (abundance) and changes in the metapopulation dynamics (occupancy) of native plants (Humphries et al., 2021; Powell et al., 2011). Hence, in response to increases in weed biomass during the initial stages of degraded grassland restoration, the relationship between aboveground biomass and species richness weakens.

### 4.3 Implication for ecological restoration projects

The findings of this study provide convincing evidence of linear relationships between aboveground biomass and plant species diversity during the initial stages of the restoration of degraded grasslands in the Xing'an League. We anticipate that these findings will provide valuable information for developing measures to enhance the efficacy of programs to restore degraded grasslands. From the perspective of carbon sequestration projects, plant diversity and biomass are two key indicators, and CCB‐based assessments can provide evidence that a given grassland project delivers tangible CCB benefits (https://www.climate‐standards.org/ccb‐standards/). These indicators can be applied to any land management project, including degraded grasslands. Based on our findings, we believe restoration initiatives that promote the recovery of degraded grasslands could potentially reach the CCB standards associated with carbon sequestration projects. In this regard, the monitoring and control of invasive weeds should be a priority during the initial stages of grassland restoration projects.

Ideally, carbon sequestration projects should meet CCB standards. To meet biodiversity requirements and avoid biodiversity, assessing the risk of weed invasion and enhancing grassland ecosystem management is necessary (Lindenmayer et al., 2012; Visseren‐Hamakers et al., 2012). Weed invasions may reduce the economic value of carbon sequestration projects. The ecological and economic benefits of controlling the density of weed species using biological control agents can be quantified. However, the risks and net benefits of biological control programs are often derived from social needs. Effective ecological management should be considered to obtain economic benefits. Studying weed invasion can also provide valuable insights for ecologists and wildlife managers interested in restoring natural ecosystems. By adopting suitable management strategies, such as controlled burning or weed control, human intervention can contribute to the sustainable maintenance of resilient ecological communities. From a population perspective, it is particularly important to gain a comprehensive understanding of the influence of invasive weeds on species that differ in biomass (Brooks et al., 2010; Catford et al., 2012; Humphries et al., 2021; Swanton & Murphy, 1996).

Grassland ecosystem management entails a diverse range of measures, including grazing, burning, hay harvesting, irrigation, fertilization, chemical treatment, and release of biocontrol agents targeting noxious weeds (Catford et al., 2012; Harker & O'Donovan, 2013; Humphries et al., 2021; Swanton et al., 2008). Extensive management typically involves the removal of a significant fraction of the existing biomass above a certain height, reducing the competition for light and maintaining the populations of those species that can persist despite the pressures associated with frequent cutting and grazing (Pavlů et al., 2007; Swift et al., 1992). Optimal management often involves the regulation of weed biomass such that the high diversity and productivity of native plant species or their preferred habitat characteristics can be promoted and maintained. In contrast, restoration is generally based on the planting/seeding of a certain number and combination of native species to restore the native structure and function of a habitat, contributing to a more rapid recovery of degraded ecosystems (Guo et al., 2018; Weidlich et al., 2020).

Linear relationships between aboveground biomass and plant species diversity can enhance the economic value of degraded grassland restoration projects (Knight & Overbeck, 2021; Liu et al., 2022; Waldén & Lindborg, 2018). Carbon sequestration programs based on degraded grassland restoration projects have provided a win–win situation between environmental conservation and increased opportunities for economic development and regional sustainability (Li et al., 2021; Tölgyesi et al., 2022). Degraded grassland restoration projects provide viable economic opportunities for carbon sequestration income (Huber et al., 2022; Kemp et al., 2013). However, strict carbon assessment standards should be established for the CCB benefits in degraded grassland restoration projects (Lindenmayer et al., 2012; Visseren‐Hamakers et al., 2012). Based on our results, creating economic incentives for carbon sequestration may positively impact biodiversity and biomass in carbon sequestration programs for degraded grassland restoration projects. Finally, models should be established to assess the economic benefits of degraded grassland restoration projects by considering biodiversity and biomass based on native and weed species.

## 5 CONCLUSIONS

Our findings, based on the assessments of the linear relationships between aboveground biomass and plant species diversity, indicate that aboveground biomass and plant species diversity could be effectively restored during the initial stages of degraded grassland restoration projects and that these findings will contribute to enhancing the successional development of degraded grassland landscapes. Weed biomass was found to have negative effects on plant species diversity and evenness in degraded grasslands, indicating that, from the perspective of promoting plant species diversity and aboveground biomass, weed biomass could serve as a key indicator of restoration performance. Moreover, effective weed control would contribute to meeting the CCB standards for carbon sequestration projects. Given a solid conceptual framework and clear vision of restoration objectives, we will be better able to address the requirements for preserving the ecosystem functions and services of degraded grassland landscapes.

## AUTHOR CONTRIBUTIONS

**Chun‐Jing Wang:** Conceptualization (equal); data curation (equal); formal analysis (equal); investigation (equal); methodology (equal); project administration (equal); resources (equal); software (equal); supervision (equal); validation (equal); visualization (equal); writing – original draft (equal); writing – review and editing (equal). **Shan‐Feng Huang:** Conceptualization (equal); formal analysis (equal); funding acquisition (equal); investigation (equal); project administration (equal); supervision (equal); writing – original draft (equal); writing – review and editing (equal). **Chu‐Ping Wu:** Investigation (equal); validation (equal); visualization (equal). **Gai‐Ni Wang:** Funding acquisition (equal); methodology (equal); validation (equal). **Lei Wang:** Methodology (equal); resources (equal); software (equal); validation (equal); visualization (equal). **Yong‐Kun Zhang:** Funding acquisition (equal); project administration (equal). **Ji‐Zhong Wan:** Conceptualization (equal); data curation (equal); formal analysis (equal); funding acquisition (equal); investigation (equal); methodology (equal); project administration (equal); resources (equal); software (equal); supervision (equal); validation (equal); visualization (equal); writing – original draft (equal); writing – review and editing (equal).

## FUNDING INFORMATION

This study was funded by the National Natural Science Foundation of China (NSFC) (No. 42207375), the National Key Research and Development Program of China (No. 2022YFF1302603), the Basic Research Project of Qinghai Province, China (No. 2020‐ZJ‐967Q) and the funds of Climate Bridge Ltd (Shanghai).

## CONFLICT OF INTEREST STATEMENT

The authors declare no conflicting interests.

## Data Availability

The data that support the findings of this study and the list of investigated plant species are available on request from the corresponding author. The data are not publicly available due to privacy or ethical restrictions. We uploaded the data as supporting material for peer review.
